# Evaluation of the Relapse Risk and Survival Rate in Patients with Hodgkin Lymphoma: A Monocentric Experience

**DOI:** 10.3390/medicina57101026

**Published:** 2021-09-27

**Authors:** Ovidiu Potre, Monica Pescaru, Alexandra Sima, Ioana Ionita, Raluca Tudor, Ema Borsi, Miruna Samfireag, Cristina Potre

**Affiliations:** 1 Department of Internal Medicine, Hematology, Victor Babes University of Medicine and Pharmacy, 300041 Timisoara, Romania; potre.ovidiu@umft.ro (O.P.); monymona87@gmail.com (M.P.); ionita.ioana@umft.ro (I.I.); borsi.ema@umft.ro (E.B.); potre.cristina@umft.ro (C.P.); 2 Hematology Clinic, Timisoara’s Emergency City Hospital, 300723 Timisoara, Romania; 3 Second Department of Internal Medicine, Diabetes, Nutrition, Metabolic Diseases and Systemic Rheumatology, Victor Babes University of Medicine and Pharmacy, 300041 Timisoara, Romania; 4 3rd Medical Clinic, Pius Brinzeu Emergency Hospital, 300041 Timisoara, Romania; 5 Department of Neurology, Victor Babes University of Medicine and Pharmacy, 300041 Timisoara, Romania; tudor.raluca@umft.ro; 6 Department of Internal Medicine, Clinical Practical Skills, Victor Babes University of Medicine and Pharmacy, 300041 Timisoara, Romania; samfireag.miruna@umft.ro

**Keywords:** Hodgkin lymphoma, relapse risk, ABVD, BEACOPP, event free survival

## Abstract

*Background and objectives:* Hodgkin lymphoma (HL) is characterized by the presence of malignant Reed Sternberg cells. Although the current curability rate in patients with HL has increased, up to 30% of those in the advanced stages and 5% to 10% of those in limited stages of the disease, relapse. According to the studies, the relapse risk in HL decreases after 2 years. The purpose of this study is to evaluate the relapse risk and event free survival (EFS) in patients with HL treated with Doxorubicin, Bleomycin, Vinblastine and Dacarbazine (ABVD), or treated with Bleomycin, Etoposide, Doxorubicin, Cyclophosphamide, Vincristine, Procarbazine, and Prednisone (BEACOPP) regimens. *Material and methods:* In an observational, consecutive-case scenario, 71 patients (median age 32 years; range 16 to 80 years) diagnosed within a 4-year timeframe were enrolled; all patients were treated according to standards of care. The average follow-up duration was 26 months. *Results:* The risk of relapse, in patients older than 40 years, decreased after 1 year, OR = 0.707 (95% CI 0.506 to 0.988), and 2 years, OR = 0.771 (95% CI 0.459 to 1.295), respectively. Patients in the advanced stages had a higher International Prognostic Score (IPS) (score ≥ 4). The overall survival at 2 years was 57.74% and the disease-specific survival at 2 years was 71.83%. Regardless, the chemotherapy regimen and the EFS time, advanced stage, high IPS and bulky disease were still associated with an increased relapse risk in patients with HL. *Conclusions:* The use of ABVD chemotherapy regimen followed by 2 years EFS was associated with a reduced relapse risk.

## 1. Introduction

Hodgkin lymphoma (HL) results from the malignant transformation of B-cells, giving rise to Reed Sternberg cells. Although the currently curability rate in HL is high, up to 30% of the patients in the advanced stages of the disease and 5 to 10% of those in a limited stage, relapse [1,2,3,4]. The time until the first relapse is commonly measured since the date of diagnosis. According to research, the risk of relapse in HL decreases after 2 years [5]. This is particularly important in patients with a high risk at diagnosis. The relapse risk assessment allows for a better monitoring of the disease and patient counseling. The monitoring of side effects has an increased significance for survival in patients diagnosed with HL disease. The long-term survival of the patients with HL was lower compared to the general population as a result of the late side effects of chemotherapy [5,6]. In some early stage HL cases, a reduced dose of Doxorubicin, Bleomycin, Vinblastine and Dacarbazine (ABVD) down to 2 cycles, followed by radiation therapy, showed a lower toxicity [7]. Both ABVD and BEACOPP have a high risk of late pulmonary toxicity which appears mainly due to Bleomycin but cardiovascular toxicity is also common (pericardial effusion, valvular calcifications). While the relapse risk decreases with time, the mortality risk caused by cardiovascular diseases and a second cancer increases [5,8,9]. The occurrence of a second cancer (mainly blood cancers, breast cancer, lung cancer and thyroid cancer) is reported as being one of the main causes of mortality for the long-term survivors, and the relative risk remains high up to 40 years after diagnosis [9,10,11].

The main aim of this study was to evaluate the relapse risk, and event free survival (EFS) in patients treated with Doxorubicin, Bleomycin, Vinblastine and Dacarbazine (ABVD), or treated with Bleomycin, Etoposide, Doxorubicin, Cyclophosphamide, Vincristine, Pro-carbazine, Prednisone (BEACOPP), adjusted for possible factors influencing the HL’s prognosis, in a special setting, with a previously unevaluated population of patients. We are aware that different populations, regarding several socio-economic, geographic, anthropometric and populational characteristics may have different responses to similar interventions. Thus, analyzing the particular response in a previously unevaluated, special-setting population will create a novelty factor and will contribute to broader population studies and meta-analysis.

## 2. Materials and Methods

### 2.1. Study Design and Patients

In an observational, consecutive-case scenario study, 71 patients diagnosed with HL within a 4-year interval were enrolled. All patients were treated according to standards of care. Stage-adapted protocols were used but therapy decisions were also made based on comorbidities.

Patients’ baseline characteristics and information regarding the treatment regimen (according to ABVD or BEACOPP protocols) were collected from patients’ medical records.

According to the disease’s severity, patients were classified as:

- HL with favorable prognosis: patients in the early stage (HL stage I or II) with no risk factors defined by GSHG/EORTC or NCIC;
- HL with unfavorable prognosis: patients in the early stage (HL stage I or II) with one or more risk factors;
- HL in advanced stage of HL (including patients in stage IIB, III and IV) [12,13].

The limited stage includes IA, IB or IIA without tumors in the chest that are at least ⅓ as wide as the chest, or tumors in other areas that are at least 10 cm across (Bulky disease). Advanced stage disease includes stages III, IV, stage I or II, with Bulky tumors and stage II with B symptoms (fever, drenching night sweats and loss of more than 10% of body weight over 6 months).

The ABVD regimen was administered every 28 days for maximum of 6 cycles: Doxorubicin 25 mg/m^2^, IV, on days 1 and 15; Bleomycin 10 mg/m^2^, IV, on days 1 and 15; Vinblastine 6 mg/m^2^, IV, on days 1 and 15; and Dacarbazine 375 mg/m^2^, IV, on days 1 and 15.

The BEACOPP regimen was repeated every 14 days for 4 to 6 cycles and included: Bleomycin 10 mg/m^2^, IV, on day 8; Etoposide 100 mg/m^2^, IV, on days 1–3; Doxorubicin 25 mg/m^2^, IV, on day 1; Cyclophosphamide 650 mg/m^2^, IV, on day 1; Vincristine 1.4 mg/m^2^, IV, on day 8; Procarbazine 100 mg/m^2^, PO, on days 1–7; and Prednisone: 80 mg/m^2^, PO, on days 1–7.

Patients in an advanced stage of the disease were further classified in different categories of risk using the International Prognostic Score (IPS) which comprises the following risk factors (for each factor present the patients received 1 point) (9): serum albumin < 4 g/dL, hemoglobin < 10.5 g/dL, male gender, age ≥ 45 years, stage 4 by Ann Arbor classification, white cell count ≥ 15,000/mm^3^ and lymphocyte count < 600/mm^3^, or < 8% of white cell count. To evaluate the patient’s performance, the Eastern Oncology Cooperative Group (ECOG) scale was used.

Based on the IPS score, the patients with advanced stage disease can be classified as follows (3): low risk (IPS 0–1), intermediate risk (IPS 2–3) and high risk (IPS 4–7).

Patient’s baseline characteristics are presented in Table 1.

### 2.2. Statistical Analysis

Overall survival (OS) was measured since the date of HL diagnosis up to the date of death by any cause. Disease-specific survival (DSS) was measured since the date of HL diagnosis until the date of death, if this occurred during treatment. The EFS was defined as the time from diagnosis to the first occurrence of disease progression or death as a first event. The causes of death were classified as either secondary to HL, the acute toxicity of the treatment, heart disease, a second cancer, other cause or unknown cause. The EFS points were pre-defined points from the diagnosis and defined periods in which the subsets of patients maintained the event-free status. The relapse risk or the disease progression risk was measured from the time of diagnosis (year 0) until the next point, namely EFS 1 and 2 years, respectively. The fatal outcomes resulting from other causes were considered concurrent risks: death related to treatment, which included all death cases due to cardiac diseases and acute toxicity; death caused by a second cancer; death caused by other causes; and by unknown causes. A Bonferroni correction was used to carry out a correction for multiple comparisons. The Kaplan–Meier estimator was used to estimate the OS and DSS. The Cox proportional hazards regression used the OS as a dependent variable to identify the potential independent prognostic factors. The statistical analysis was carried out using the SPSS v20.0.

## 3. Results

The median follow-up for the surviving patients was 26 months. Of the patients, 33.9% (*n* = 24) were included in stages I and II of disease – stage I, *n* = 6 (8.5%); and stage II, *n* = 18 (25.4%)), respectively –; and 66.1% (*n* = 47) in the advanced stages – stage III, *n* = 14 (19.7%); and stage IV, *n* = 33 (46.4%)). Ten patients (14.1%) received radiotherapy followed by chemotherapy. Fifty patients (70.4%) were treated with ABVD, nine patients (12.7%) with BEACOPP and two patients (2.8%) with escalated BEACOPP. The patients in the advanced stages, representing 47 patients (66.1%), had a high IPS score (score ≥ 4).

### 3.1. Relapse Risk

The relapse risk was calculated at the time of HL diagnosis, and it was evaluated using a multivariate regression analysis correlated with the negative prognostic factors. From the time of diagnosis, most of the future events were the result of lymphoma relapse. Thus, it was noticed that the risk of relapse increased in both patients in the advanced stages of the disease at all established moments and in patients with a high IPS score. The risk of relapse, in patients older than 40 years, decreased after 1 year, OR = 0.707 (95% CI 0.506 to 0.988), and after 2 years, OR = 0.771 (95% CI 0.459 to 1.295), respectively. The risk of relapse was similar for men and women. Significant differences in the relapse risk increases were observed between the studied time points (Year 0, EFS at 1 year and EFS at 2 years) (Table 2).

### 3.2. Overall Survival of Patients with HL

Of the 71 patients, 30 died, and 20 of them (66.66% of the total deaths) were caused by HL (Table 3). For all patients, from the time of diagnosis, the 2-year OS rate was 57.74% and the 2-year DSS rate was 71.83%.

To evaluate possible predictors for prognosis, multivariate Cox regression analysis models were built, demonstrating that the following were associated with increases in death risk: an age of between 45 and 60 years, HR = 1.014 (95%CI: 0.991 up to 1.037); *p* < 0.05,, and advanced stage, HR = 1.089 (95%CI: 0.787 up to 1.507), *p* < 0.05, and low performance status, HR = 1.44 (95%CI: 1.17 up to 2.32); *p* < 0.05.

## 4. Discussion

In this study, the characterization of the risk of relapse in patients with HL treated with ABVD or BEACOPP chemotherapy was made based on the criteria of not having any events at the time points assigned (1 and 2 years) [12,13]. Advanced stage, high IPS, and bulky disease were associated with a risk of relapse after 1Y-EFS and 2Y-EFS, while age >40 and male gender did not have a prognostic impact.

For patients with EFS the 2-year OS and DSS rates were over 90%. Although the age represented an important prognostic factor for DSS and OS, age did not significantly influence EFS. Although we cannot exclude the impact of the small cohort, as there were only 71 patients, this suggests that, provided that an older patient can receive ABVD chemotherapy for curative purposes, the risk of relapse is similar to that of younger patients. A lower OS probably reflected that older patients who relapsed were not usually eligible for high-dose chemotherapy/autologous stem cell transplantation (HDC/ASCT) and could face additional toxicity from the overlapped therapy of other diseases and the biology of aging.

Consistent with other studies, despite the improved relative survival, an increased mortality risk was noticed in all age categories and the most obvious risk was found in younger patients (aged between 20 and 29 years and 30 and 39 years). It must be highlighted that, for the general population from this age category, the mortality risk is low and, consequently, any risk among the HL survivors increases. A relatively higher mortality risk is caused by a second cancer, an d by cardiovascular diseases, leading to a higher risk for other diseases. HL was reported in many studies in patients evaluated for more than 20 years [5,9,11,12] and no improvement in OS was identified.

Unlike our findings, a study regarding HL reported that the patients diagnosed with limited stages of the disease who obtained EFS at 24 months, showed a survival rate comparable with that of the general population [13,14,15]. Furthermore, a study performed on 602 patients diagnosed with early stage HL, who followed ABVD treatment and further radiation therapy, revealed a 3-year progression-free survival rate of 94.6% [16]. These data regarding the risk and time of the relapse have significant clinical implications. We consider that the aggressive post-treatment imagistic evaluation in HL is not justified, because of two reasons. First, for the patients in the limited stages of the disease who do not have any event at 2 years, the relapse risk is low (results are comparable with other studies) [17], and second, there is a high probability of achieving a false positive CT scan at the long-term periodical radiologic reevaluation. Additionally, we revealed the importance of focusing on the symptoms reported by the patient [18,19]. Additionally, our data supported using the 2-year EFS as a final point for clinical studies that were designed to reevaluate the first line treatment protocols, as well as ASCT protocols.

This study has the advantage to evaluate the HL’s prognosis and predictors for 2-year outcomes in a special setting in a previously unevaluated population.

This analysis has some limitations. The main goal of the risk analysis was to evaluate the relapse risk, but the probability of other events (for instance cardiac disease as a cause of death) varies among the patients. Our study included a small number of patients and larger trials are needed for further conclusions.

## 5. Conclusions

Regardless, the chemotherapy regimen and the EFS time, advanced stage, high IPS and bulky disease are still associated with an increased relapse risk in patients with HL. Male gender and higher age were associated with a reduced relapse risk in patients treated with ABVD chemotherapy regarding the 2-year EFS analysis. Although the relative survival improved with the EFS duration for all patients, it remains inferior compared to the general population and suggests that an increased risk in mortality persists in patients with HL, regardless the choice of treatment.

## Figures and Tables

**Table 1:** Patient’s baseline characteristics.

Characteristic	No. of Patients (%)
Male gender	45 (63.4)
**Age, years (y)**	
<20 y	4 (5.6)
20–29 y	16 (22.5)
30–39 y	16 (22.5)
40–49 y	9 (12.7)
50–59 y	11 (15.5)
60–70 y	14 (19.7)
>70 y	1 (1.4)
**Treatment approach**	
Limited	24 (33.9)
Advanced	47 (66.1)
Radiotherapy received	
Yes	10 (14,1)
No	61 (75.9)
Chemotherapy for primary therapy	
ABVD * + radiotherapy	10 (14.1)
ABVD	50 (70.4)
BEACOPP **	9 (12.7)
BEACOPP escalated	2 (2.8)
**Stage**	
I	6 (8.5)
II	18 (25.4)
III	14 (19.7)
IV	33 (46.5)
Bulky disease ≥ 10 cm	19 (26.8)
B symptoms	31 (43.66)
**Performance status**	
0–1	59 (83.09)
≥2	12 (16.91)
IPS *** §	
Low 0–1	60 (84.5)
High ≥ 4	11 (15.6)
Histology	
Mixed cellularity	28 (39.4)
Nodular sclerosis	39 (54.9)
Lymphocyte rich	4 (5.6)

* ABDV = treatment with Doxorubicin, Bleomycin, Vinblastine and Dacarbazine, ** BEACOPP = treatment with Bleomycin, Etoposide, Doxorubicin, Cyclophosphamide, Vincristine, Pro-carbazine, Prednisone, *** IPS = International Prognostic Score

**Table 2:** Factors associated with the relapse risk.

Group	Year 0	EFS ^1^ at 1 Year	EFS at 2 Years	* *p*-Value
Advanced stage				
Relapse risk, (OR 95% CI)	1.707 (1.036 to 2.811)	1.299 (0.781 to 2.160)	1.508 (0.629 to 3.619)	0.029
High IPS ^2^				
Relapse risk, % (95% CI)	2.1414 (1.844 to 2.455)	1.688 (1.022 to2.448)	1.333 (0.336 to 1.555)	0.028
Age > 40 years				
Relapse risk, (OR 95% CI)	1.011 (0.727 to 1.407)	0.707 (0.506 to 0.988)	0.771 (0.459 to 1.295)	0.050
Male gender				
Relapse risk, (OR, 95% CI)	0.964 (0.333 to 2.794)	0.795 (0.277 to 2.281)	0.453 (0.075 to 2.745)	0.077
Bulky disease				
Relapse risk, (OR, 95% CI)	1.966 (0.608 to 6.358)	1.355 (0.418 to 4.387)	1.040 (0.170 to 6.348)	0.040

* The presented *p*-values are describing the statistical significance of differences between columns. ^1^ EFS = event free survival, ^2^ IPS = International Prognostic Score.

**Table 3:** Causes of death.

Hodgkin Lymphoma	20 (66.66)
Acute treatment toxicity	2 (6.66)
Second cancer	6 (20.02)
Cardiac disease	2 (6.66)

## Data Availability

Restrictions apply to the availability of these data. Data was obtained from the integrated hospital information system’s database of Timisoara’s Emergency City Hospital and are available from authors with the permission of the hospital’s Board of Executives. All requests should include rationale for analysis and pre-specified statistical analysis plan.
